# The 21st Century Agriculture: When Rice Research Draws Attention to Climate Variability and How Weedy Rice and Underutilized Grains Come in Handy

**DOI:** 10.3390/plants9030365

**Published:** 2020-03-16

**Authors:** Noraikim Mohd Hanafiah, Muhamad Shakirin Mispan, Phaik Eem Lim, Niranjan Baisakh, Acga Cheng

**Affiliations:** 1 Functional Omics and Bioprocess Development Laboratory, Institute of Biological Sciences, Faculty of Science, University of Malaya, Kuala Lumpur 50603, Malaysia; 2 The Centre for Research in Biotechnology for Agriculture, University of Malaya, Kuala Lumpur 50603, Malaysia; shakirin@um.edu.my; 3 Institute of Ocean and Earth Science, University of Malaya, Kuala Lumpur 50603, Malaysia; phaikeem@um.edu.my; 4 School of Plant, Environmental, and Soil Science, Louisiana State University Agricultural Center, Louisiana State University, Baton Rouge, LA 70803, USA

**Keywords:** climate change, food security, green revolution, modern rice, underutilized grains, weedy rice

## Abstract

Rice, the first crop to be fully sequenced and annotated in the mid-2000s, is an excellent model species for crop research due mainly to its relatively small genome and rich genetic diversity. The 130-million-year-old cereal came into the limelight in the 1960s when the semi-dwarfing gene *sd-1*, better known as the “green revolution” gene, resulted in the establishment of a high-yielding semi-dwarf variety IR8. Deemed as the miracle rice, IR8 saved millions of lives and revolutionized irrigated rice farming particularly in the tropics. The technology, however, spurred some unintended negative consequences, especially in prompting ubiquitous monoculture systems that increase agricultural vulnerability to extreme weather events and climate variability. One feasible way to incorporate resilience in modern rice varieties with narrow genetic backgrounds is by introgressing alleles from the germplasm of its weedy and wild relatives, or perhaps from the suitable underutilized species that harbor novel genes responsive to various biotic and abiotic stresses. This review reminisces the fascinating half-century journey of rice research and highlights the potential utilization of weedy rice and underutilized grains in modern breeding programs. Other possible alternatives to improve the sustainability of crop production systems in a changing climate are also discussed.

## 1. Introduction

The blueprint to achieve a more sustainable future for all, or better known collectively as the sustainable development goals (SDGs), was developed by the United Nations in 2015 as a universal call for action to protect the earth, end poverty, and ensure that humans live in peace and prosperity [1,2]. Agriculture, the largest user of natural resources like water and land in the world, plays a direct role in achieving some of the 17 developed SDGs, especially in terms of water, biodiversity, climate change, poverty, sustainable energy, and cities [3]. The green revolution (GR) succeeded in increasing crop production after the mid-20th century and saved millions of lives [4]. However, a new paradigm of green agriculture, where less resources are used to grow crops, is required in the current century to feed the ever-growing population amid climate change. The Fifth Assessment Report prepared by the Intergovernmental Panel on Climate Change in 2014 stated that crop yield in low-latitude countries would be consistently and negatively affected by climate change. The average global temperature increased by ~0.13 °C since the 1950s and is expected to grow at a faster pace (~0.2 °C per decade) in the next several decades [5]. The increment in maximum temperature in certain locations may affect the yield and reproduction of many important crops [6]. For instance, a one-degree increase in the maximum temperature in Nepal caused a decrease in rice production to an average of about 130 kg/ha [7]. A more coherent and systematic approach to global food production is, therefore, crucial for sustainable agriculture in the 21st century [8,9].

The true grass family Poaceae (or Gramineae) is long considered as the most economically important plant family for food production, comprising more than 10,000 species, which include the “big three” cereals—wheat (*Triticum aestivum*), maize (*Zea mays*), and rice (*Oryza sativa*) [10]. Rice, with over 40,000 distinct varieties grown on every continent except Antarctica [11,12], is the most important food crop in the developing world [13,14]. It is a dependable staple for more than half of the entire world’s population, including about 550 million undernourished people living in Asia [15,16]. The genus *Oryza*, which emerged almost 130 million years ago, consists of 22 wild and two cultivated species, namely *O. sativa* and *O. glaberrima* [17]. Pericarp color, dormancy, shattering, panicle architecture, and tiller number are among the primary traits used to differentiate between the wild and cultivated species [18]. The wild rice *O. rufipogon*, commonly known as Asian rice, is the recognized progenitor of *O. sativa* that contains two major subspecies: long-grain, non-sticky *indica* rice and short-grain, sticky *japonica* rice [19]. Based on a geographical analysis, *indica* rice was domesticated in the Himalayas, likely eastern India, while *japonica* rice was domesticated in southern China [20]. The African cultivated rice *O. glaberrima*, on the other hand, is grown in small areas in West Africa [21].

The old saying “rice is life” reflects the importance of this ancient grain to humankind not only as a staple food but also as cultural and spiritual sustenance [22]. Through the lens of science, rice is an excellent model species for plant biology research, particularly for studies on monocotyledonous plants, due to its relatively small genome size of 430 Mb [23,24,25]. It is the first crop to be fully sequenced, furnishing a valuable reservoir of genetic variation for numerous agriculturally important traits such as yield and stress tolerance [18]. *Oryza* species were classified into three main groups (or complexes), called the primary, secondary, and tertiary gene pools, based mainly on the ease of gene transfer into cultivated species [11,23,26]. The primary gene pool (*O. sativa* complex) consists of Asian cultivated rice (*O. sativa*), weedy rice (*O. sativa f. spontanea*), wild ancestor species (*O. rufipogon* and *O. nivara*), and other AA-genome variant species. The *O. sativa* complex constitutes primarily the diploid AA-genome species (2n = 24) with perfect synapsis and relatively high sexual compatibility, and pollen and panicle fecundity of F1 hybrids [21]. The secondary gene pool (*O. officinalis* complex) encompasses other non-AA-genome species, whereas the tertiary gene pool (*O. meyeriana* and *O. ridleyi* complex) consists of species of other genera in the tribe Oryzeae [27].

The past half-century witnessed a handful of eminent scientific innovations for agricultural systems, from the development of high-yielding semi-dwarf varieties of various major crops through systematic breeding programs to more sophisticated studies of plants at the molecular level, with the latest innovation being the clustered regularly interspaced short palindromic repeats (CRISPR)/CRISPR-associated protein 9 (Cas9) gene-editing technology [28,29]. In rice specifically, its first completed public genome not only contributed to significant advancements in its genetics and breeding but also paved the way for the sequencing of more complicated crop genomes such as wheat and maize [30]. Nonetheless, the fact that the global demand for rice is continually increasing while its production per capita is decreasing makes it necessary for researchers to constantly look for critical ways to further improve the crop. Although one of the notable challenges in rice production is the presence of weedy rice [31], recent studies suggested that weedy rice has novel sources of resistance to devastating rice diseases such as sheath blight (caused by *Rhizoctonia solani*) and blast (caused by *Magnaporthe oryzae*) that cause severe crop losses worldwide [32]. In this review, we attempted to synthesize the past research on rice biology and genetics and highlight the main gaps and future directions in rice research. We also discussed the potential utilization of weedy rice and underutilized grain crops in the development of climate-resilient rice varieties.

## 2. Highlights of Rice Research since the Green Revolution

The GR in the 1960s resulted in the development of IR8, the first semi-dwarf, high-yielding variety (HYV) of rice by the International Rice Research Institute (IRRI). The seeds of the IR8, hailed as “miracle seeds”, were credited with saving millions of lives in many famine-prone countries, particularly those in Asia such as India and China [33]. Nevertheless, its reliance on heavy doses of fertilizers and irrigation to maximize yield sparked controversy for decades [34]. As the 21st century heralds a new GR, it is essential to dwell on the past achievements and failures during the early and late GR to make sure that all critical aspects of crop improvement are thoroughly considered for the next, greener revolution.

### 2.1. Early Green Revolution

The discovery of the semi-dwarfing (*sd-1*) gene by the late Norman E. Borlaug, a Nobel Peace Prize Laureate who is known as the Father of GR, dramatically enhanced the development of HYV throughout the world, remarkably for the big three cereals. The semi-dwarf trait became credible in supporting the heavy grains of HYVs and preventing the plants from lodging. Between 1966 and 1986, short-statured rice varieties adopted approximately 60% of the global rice land [35]. The first HYV IR8 was derived from the cross between Dee-geo-woo-gen (DGWG), a dwarf Chinese variety with the *Sd-1* gene and Peta variety from Indonesia which is tall, vigorous, and good in taste [33]. It was released in 1966, and quickly became the most planted rice variety in some areas of Asia. Although the IR8 has some remarkable traits such as lodging resistance and good fertilizer response, it also possesses several drawbacks, with the major ones being its long growth duration (i.e., matures in 130 days) and susceptibility to many diseases and insects. Thereupon, the breeding programs at IRRI focused mainly on the development of short-duration and/or multiple disease- and insect-resistant varieties, leading to the release of ~30 IR varieties by the mid-1980s [35]. In addition to having a considerably short growth cycle, newly developed modern rice varieties such as IR36, IR50, and IR64 are photoperiod-insensitive and can be planted at any time of the year [36].

The success of the IR8 was recognized globally by breeders working on rice and beyond. Semi-dwarf varieties were widely used as the donor parent in many intensive breeding programs for other major food crops such as wheat [37] and maize [38]. The modern varieties, by and large, respond better to nitrogen fertilizer compared to the traditional varieties, which usually grow excessively tall, lodge early, and produce tiller extensively with low yields [36]. However, it is important to note that the production of the modern varieties requires the utilization of a substantial amount of chemical fertilizers and pesticides, with the adoption of efficient irrigation systems to boot [39,40]. Another major issue of growing modern varieties is the increase in monoculture, continuous cultivation of a uniform crop variety on a particular land, which reduces the genetic diversity of crops and agricultural system, thus increasing the crop vulnerability to agricultural risks, notably disease and pest infestation [1,34]. Monoculture is now dominant in many countries, especially those that benefited from GR.

### 2.2. Late Green Revolution

The global production of wheat, maize, and rice in many parts of the world increased regularly since the 1960s, and it nearly doubled within a mere two decades that consequently reduced famine and hunger crises [41]. Between 1980 and 2000, the world population grew from 4.4 billion to 6.1 billion, with more than 90% of the growth occurring in developing countries. The agricultural areas in these countries grew from 2.85 billion ha in 1980 to 3.17 billion ha in 2001 [42]. The production of rice in the year 2000 increased by more than 200% in certain countries. Since the release of the IR8 variety in 1966, ~70% of world’s rice land was planted with HYV during the mid-1990s, and their distinctive characteristics include higher yield potential, improved grain quality, shorter growth duration, and resistance to multiple diseases and insects [36].

The late GR saw tremendous improvement in the efficient use of molecular and cellular approaches in rice research. Genetic engineering in rice began way back in the 1980s, with the first transgenic rice reported in the late 1980s [43,44]. Significant advancements in the genetic transformation of rice were made since then, with numerous gene transfer protocols with appropriate promoters, markers, and reporter genes being developed and employed to introgress foreign genes into rice. Standardized protocols for the production of transgenic rice of more than 60 rice varieties that include *indica*, *japonica*, *javanica*, and elite African cultivars are also reportedly available [45]. Much focus was given to developing rice with resistance toward insects [46], pests [47], viruses [48], and diseases such as sheath blight [48] and bacterial leaf blight [49]. One renowned example is the genetically engineered, insect-resistant Bt rice which was developed by introducing the insecticidal genes from *Bacillus thuringiensis* Berliner (Bt) into rice [50]. Although Bt rice showed good resistance to yellow and striped stem borer, both in laboratory and in field conditions, its commercial planting was long delayed due to regulatory restrictions for food safety concerns [51]. While the development of transgenic rice focused mainly on insect and disease resistance during the 1990s, the most remarkable success story at that time is perhaps the development of beta-carotene-producing golden rice [52], a nutritionally enhanced genetically modified crop which was only recently approved safe for human consumption in the Philippines after obtaining food safety approval from Australia, New Zealand, and the United States [53,54].

Rice research continued to grow and flourish as it entered the new millennium, taking its improvement far beyond the conventional practice limits. With the development of linkage and qualitative trait locus (QTL) maps, marker-assisted selection (MAS) is the most common approach used internationally. This is particularly the case for developing high-yielding rice with improved resistance against biotic and abiotic stresses, which was one of the primary goals to improve global rice production during the late era of GR [11,55]. Most successful examples of MAS include the development of rice introgressed with *Xa* genes for bacterial blight resistance and Sub1A for submergence tolerance [56]. Genome-wide association studies (GWAS) represent another powerful tool used to dissect the genetics and identify markers associated with complex traits in rice, including flowering time, plant height, grain yield, and grain shape for use in MAS [57,58].

### 2.3. 21st Century

The completion of the rice genome in the mid-2000s marked a momentous milestone in rice research, opening seemingly endless doors for gene discovery not only in rice but also in other crops [59,60]. Rice, together with thale cress (*Arabidopsis thaliana*) that had its genome completed in 2001 [61], are the best-characterized model species in plant biology [23,62]. Nevertheless, rice is a C3 crop that has considerably lower photosynthetic efficiency than C4 crops such as maize and sorghum [63]. Much research was devoted to engineering C4 photosynthetic traits into rice, which could increase its yield up to 50% while using half the water. During the last decade, more than 20 comparative transcriptomic studies were published with the identification of potential C4 genes and their regulatory mechanisms [64]. This was made possible by advances in next-generation sequencing technologies, gene discovery, and, more recently, genome editing platforms [65].

**Figure 1:**
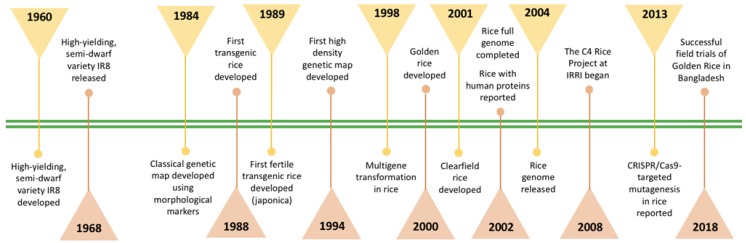
Milestones in rice research since green revolution.

At present, there are four major tools for genome editing, which include zinc finger nuclease (ZFN), transcription activator-like effector nuclease (TALEN), meganuclease, and the latest one being the CRISPR/Cas system. CRISPR/Cas system, which utilizes the adaptive mechanism of prokaryotes toward foreign deoxyribonucleic acid (DNA) fragments, successfully generated mutagenesis in transgenic rice [66]. The past decade saw a noticeable increase in the application of CRISPR/Cas genome editing in plant research, especially after the successful expression of the system in two monocot (rice and sorghum, *Sorghum bicolor*) and dicot (thale cress and tobacco, *Nicotiana tabacum*) plants [67]. The system was utilized for multigene knockouts in plants, for example, targeted mutagenesis of paralogous cyclin-dependent kinase (*CDK*) genes in rice [68]. The study conducted by Shan et al. [28] proved that the CRISPR/Cas system was a rapid method for gene targeting in rice protoplasts (within 1–2 weeks) for generating mutated rice plants (within 13–17 weeks). Currently, the CRISPR/Cas9 system is widely used to edit genes associated with yield, quality, and disease resistance in rice. The important milestones in rice research since the GR are displayed in Figure 1.

## 3. Weedy Rice and Underutilized Grain Crops as Potential Complement to Existing Rice Research

The 21st century witnessed increasing attention among researchers in laying a strong foundation for a greener revolution, where improved crop varieties require less inputs, especially water and fertilizer, to feed the estimated 9.8 billion people by the mid-century [69,70]. Cantrell and Hettel [71] highlighted that rice research in the 21st century should emphasize how to reduce both the production and the research gaps, along with strategic research plans to develop and utilize new technologies and tools. With the constant rise in food demand and rapid changes in consumption patterns, radical research approaches are crucial to complement fundamental exploration in improving both major and underutilized (or orphan and neglected) plant species [72,73]. In fact, the past decade saw the emergence of multiple studies on the lesser known plants as one of the prime strategies in strengthening the four pillars of food security, which include the availability, access, utilization, and stability of food [1,74].

In the recent past, unique research trends were observed in many rice improvement programs globally, from uncovering the worth of the undesirable weedy rice to unearthing the potential of underutilized crops in achieving sustainable rice production. Weedy or obnoxious red rice, known as the unwanted plants of *Oryza*, was recently reported to possess novel sources of stress tolerance or resistance, although its presence can lead to the reduction of both the quantity and quality of the cultivated grains [32]. Evolved as an intermediate between the wild and cultivated species, weedy rice generally exhibits a high competitive ability against cultivated rice for resources and it is considered a serious threat to rice production in many major rice-producing countries [75]. Ironically, the competitive ability and adaptive evolutionary traits of weedy rice such as stress tolerance, increased seed dispersal, and dormancy [76,77,78,79] could be useful to maximizing resource use efficiency and yield of rice amidst the current rapid climate uncertainties. The study conducted by Ziska et al. [80] demonstrated that weedy rice responded positively to elevated temperature and carbon dioxide (CO_2_) concentration, showing height increase with greater tiller and panicle formation.

Apart from having resistance to abiotic stresses, weedy rice also displays a high degree of resistance toward certain biotic stresses, such as rice blast and sheath blight caused by *Magnaporthe oryzae* and *Rhizoctonia solani*, respectively [32]. A total of 28 QTLs associated with blast resistance were identified from two weedy rice ecotypes present in the United States, namely, black hull awned and straw hull awnless [81]. Furthermore, the tallness of weedy rice helps it to avoid damage by sheath blight disease that causes injury to rice stem, leaf, and sheath [32]. Table 1 summarizes some important genes linked to biotic and abiotic stresses in weedy rice. Exploiting the full potential of weedy rice, especially its gene pools, can be beneficial for breeding and evolutionary studies of modern rice [82]. The virtue of weedy rice is finally deliberated, and this is most likely driven by the increased knowledge and awareness on the adverse effects of climate change.

Urbanization is one of the most dominant demographic trends, with approximately 70% of the world’s population projected to live in cities by the mid-century [89]. In urban environments, dietary habits and meal patterns can vary significantly between the rich, the middle class, and the poor communities. With varying diet regimes among the urban communities especially those in developed nations, the challenge of fulfilling consumer needs and demands becomes bigger than ever [90,91,92]. Many researchers today would agree that the development of underutilized crops that feed only certain communities is equally important as the improvement of common staple crops such as rice that feed the majority [72,93,94]. This perhaps explains why the research on underutilized crops gained momentum in the current era. Not only are these crops important in materializing a diversified food basket, but they are also valuable genetic resources for breeding programs of major crops and maintaining global biodiversity [34,95].

**Figure 2:**
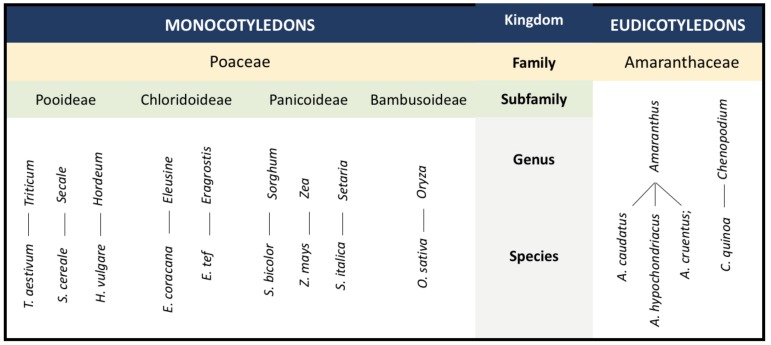
Simplified phylogenetic relationship between selected crops in the Poaeeae and Amaranthaceae families modified from References [106,107]).

A group of long-overlooked ancient grain crops, such as teff (*Eragrostis tef*), quinoa (*Chenopodium quinoa*), and amaranth (*Amaranthus* spp.) to name a few, finally received the research attention that they deserve in the last couple of years due mainly to their hardiness, versatility, and exquisite nutritional benefits [1,92,95,96]. These underutilized crops were a staple in their native homes for hundreds of years, and they possess some degree of tolerance to certain stresses, as shown in Table 2 [97,98,99,100,101,102,103,104]. An evidential example of their superior genes of nutrional importance is the development of protato (protein-rich potato) that was engineered to express the *AmA1* albumin protein of *Amaranthus hypochondriacus* [105]. This suggests that the genetic and genomic resources of such potential underutilized crops can be exploited to improve rice cultivars through identification and transfer of desirable alleles or traits. A simplified phylogenetic relationship between the discussed grain crops is presented in Figure 2.

## 4. Laying the Route to Sustainable Rice Production: What Can We Possibly Do?

**Figure 3:**
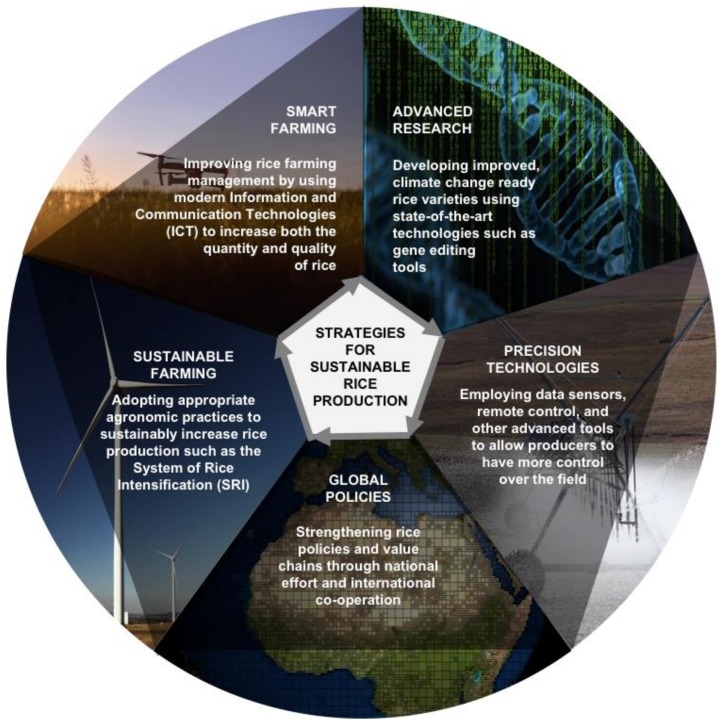
Plausible strategies to achieve sustainable rice production.

The principal aim of sustainable crop production is to optimize production by sustainably managing biological processes, biodiversity, and ecosystem services, while considering the key factors, such as economic, political, social, and environmental effects [108]. In a narrower sense, sustainable rice production is achieved when production per unit area increases as a result of ecologically regenerative approaches that integrate biodiversity and soil health rather than excessive utilization of inputs such as chemical fertilizers and pesticides [109]. Figure 3 presents several strategies which can potentially contribute significantly to sustainable rice production. It is important to ensure that the strategies used will offer socio-economic benefits to producers, both large- and small-scale, and to society for all social classes.

According to Gerber [110], a sustainable agricultural system is based mainly on the prudent use of both recyclable and renewable resources. By contrast, a system that depends on finite natural resources cannot be sustained indefinitely. The use of renewable resources (such as wind, solar, and biomass energy) to grow rice is generally still limited [111]. One of the major barriers to adopting these technologies is capital and/or construction costs, which could be overcome by implementing renewable energy subsidies to rice farmers. The promotion and utilization of renewable resources in rice fields can help promote long-term environmental stewardship, especially in relation to conserving soil quality, the main factor influencing rice production [112]. Sustainable rice production can also be supported by other means such as simplified and reduced-input farming practices. A dynamic rice production system should allow producers to choose and adopt the best combinations of practices based on their local environmental conditions and production constraints, achieving high levels of output with minimal inputs. One notable example is the system of rice intensification (SRI) (Figure 3), which recommends some sustainable agronomic practices such as application of compost or organic fertilizers and draining extra water in order to keep rice fields in saturated, non-flooded conditions [113].

Genetically improved grain crops accounted for an increase in yield of more than 50% in recent decades, and plant breeders must achieve similar or better results to feed the growing population [114]. Rice should continue to be improved along with those rising underutilized crops. With the many potential future effects of global warming, rice breeders need to develop a genetically diverse portfolio of improved cultivars that are well suited to a wide range of farming practices and agro-ecosystems [115]. During the last century, about three-quarters of crop genetic diversity disappeared; hence, increased support in collecting and conserving genetic resources is much needed [116]. Crop diversification is one of the crucial ways to preserve the genetic diversity. However encouraging the substitution of common crop staples with lesser known crops certainly does not happen overnight in every part of the world [1].

A suitable complement to sustainable farming is smart farming, where automated and connected agriculture are applied. Smart farming enables sophisticated field management by integrating advanced technologies, such as unmanned aerial vehicles (UAVs), artificial intelligence (AI), and Internet of things (IoT) into existing farming practices [117]. The utilization of different sensors and connected devices in smart farming are tailored specifically to optimize the quality and quantity of inputs, while preserving resources, from delivering visibility into crop and soil health to predicting crop performance and detecting outbreaks of harmful pests [118]. For rice production, smart farming recently became routine in some countries (such as the United States and Japan) that can afford the high cost of technology [119]. A more cost-effective and flexible smart farming system is pivotal to attract more rice-producing countries to adopt this farm management concept in the near future. It is feasible that small- to medium-sized farming operations could begin by implementing precision farming technologies that monitor and analyze the needs of individual crops and fields. Unlike smart farming (which involves connected technologies that link to all farm operations), precision technologies focus on precise measurements using individual sensors or devices, thus offering economic flexibility that is easier to establish [120].

To encourage low- and medium-income rice producers to engage in sustainable farming practices, many of the current agricultural policies will need to be revised. New policies should eliminate any existing subsidies that drive producers to overuse resources [121]. For example, incentives that encourage the use of fertilizers need to be removed [122]. Alternatively, policymakers could provide incentives for producers to utilize natural resources wisely. It is important for policymakers to commit to engaging with and transferring knowledge to producers, in the interest of supporting the improvements in their livelihoods and in social conditions. The gap between sustainable agricultural policies and how they are perceived should be identified, and a clear approach on how to adopt these policies should be defined.

Undoubtedly, rice, being one of the world’s major crops with a small genome size, garnered plenteous attention from the scientific community [123]. Unfortunately, this crop suffered a loss in genetic diversity, especially after the GR, where monoculture farming that relies heavily on chemical inputs began to monopolize most croplands [124]. It was reported that yields in many major rice-producing countries such as China is plateauing, and the yield gap between rice fields (actual yield) and research stations (potential yield) is still an ongoing issue in many countries [125]. Closing this gap is essential and requires collaborative efforts between breeders and governments to ensure that rice production continues to increase in a sustainable manner. Major investment for rice research is needed to revitalize breeding programs and technology transfer schemes in developing countries to provide producers with improved varieties and the knowledge of appropriate technologies, as well as to enhance their skills through suitable programs, such as farmer field schools.

## Figures and Tables

**Table 1:** Examples of important genes linked to biotic and abiotic stresses in weedy rice.

Gene(s)	Biotic or Abiotic Stress	Reference
*Asr1*	Salinity stress	[83]
*Bar*	Basta herbicide	[84]
*EXPA3*	Salinity tolerance	[85]
*HKT, NHX1* and *SOS1*	Salinity stress	[86]
*OVP1*	Cold stress	[87]
*PDR8*	Non-host resistance	[83]
*Pi-ta* and *Ptr(t)*	Blast	[88]
*Rc, Bh4* and *Phr1*	Aging	[31]
*Snl6*	Bacterial blight	[83]

**Table 2:** Fundamentals and important attributes of potential underutilized grains.

	Cereal		Pseudo-cereal	
	Teff	Proso Millet	Quinoa	Amaranth
**Centre of diversity**	Eastern Africa	China	Latin America	South America
**Family**	Poaceae	Poaceae	Amaranthaceae	Amaranthaceae
**Cultivated species**	*Eragrostis tef*	*Panicum miliaceum*	*Chenopodium quinoa*	*Amaranthus caudatus*; *A. cruentus*; *A. hypochondriacus*
**Genome size**	ca. 730 Mbp	ca. 1020 Mbp	ca. 1450 Mbp	ca. 500 Mbp
**Chromosome number**	2n = 4x = 40	2n = 4x =36	2n = 4x =36	2n = 2x = 32 or 2n = 2x =3 4
**Photosynthetic pathway**	C4	C4	C3	C4
**Salinity tolerance**	Broad intraspecific variation	Tolerant	Tolerant	Tolerant
**Cold tolerance**	Tolerant	Sensitive	Tolerant	Sensitive
**Drought tolerance**	Moderately tolerant	Tolerant	Tolerant	Tolerant
**Heat tolerance**	Tolerant	Tolerant	Tolerant	Tolerant
**Waterlogging tolerance**	Tolerant	Sensitive	Sensitive	Sensitive

Sources: [1,97,98,99,100,101,102,103,104]).
